# Lectins with Anti-HIV Activity: A Review

**DOI:** 10.3390/molecules20010648

**Published:** 2015-01-06

**Authors:** Ouafae Akkouh, Tzi Bun Ng, Senjam Sunil Singh, Cuiming Yin, Xiuli Dan, Yau Sang Chan, Wenliang Pan, Randy Chi Fai Cheung

**Affiliations:** 1 Department of Biology and Medical Laboratory Research, Faculty of Technology, University of Applied Sciences Leiden, Zernikdreef 11, 2333 CK Leiden, The Netherlands; E-Mail: ouafae@live.nl; 2 School of Biomedical Sciences, Faculty of Medicine, The Chinese University of Hong Kong, Shatin, Hong Kong, China; E-Mails: ycm1907@gmail.com (C.Y.); s1155009788@mailserv.cuhk.edu.hk (X.D.); bomberharo@gmail.com (Y.S.C.); b132588@mailserv.cuhk.edu.hk (W.P.); chifaicheung@cuhk.edu.hk (R.C.F.C.); 3 Department of Biochemistry, Manipur University, Canchipur, Imphal 795003, India; E-Mail: sunil_senjam@rediffmail.com

**Keywords:** lectins, anti-HIV, worm, cyanobacteria, actinomycete

## Abstract

Lectins including flowering plant lectins, algal lectins, cyanobacterial lectins, actinomycete lectin, worm lectins*,* and the nonpeptidic lectin mimics pradimicins and benanomicins, exhibit anti-HIV activity. The anti-HIV plant lectins include *Artocarpus heterophyllus* (jacalin) lectin, concanavalin A, *Galanthus nivalis* (snowdrop) agglutinin-related lectins, *Musa acuminata* (banana) lectin, *Myrianthus holstii* lectin, *Narcissus pseudonarcissus* lectin, and *Urtica diocia* agglutinin. The anti-HIV algal lectins comprise *Boodlea coacta* lectin, *Griffithsin*, *Oscillatoria agardhii* agglutinin. The anti-HIV cyanobacterial lectins are cyanovirin-*N*, scytovirin, *Microcystis viridis* lectin*,* and microvirin. Actinohivin is an anti-HIV actinomycete lectin. The anti-HIV worm lectins include *Chaetopterus variopedatus polychaete* marine worm lectin, *Serpula vermicularis* sea worm lectin, and C-type lectin Mermaid from nematode (*Laxus oneistus*)*.* The anti-HIV nonpeptidic lectin mimics comprise pradimicins and benanomicins. Their anti-HIV mechanisms are discussed.

## 1. Introduction

In the last few decades, Africa remains the continent which has been afflicted to the most serious extent by the HIV/AIDS pandemic, although the number of infections elsewhere, for instance in Asia, has also been on the rise [1]. Many children die from relatively preventable causes, especially in places where there is a high incidence of HIV infections/AIDS [2]. The search for plant extracts with anti-HIV activity is continuing [3]. Polysaccharides [4] and other compounds [5] have been proposed or used for treatment. Lectins have been extensively studied because they possess a variety of potentially exploitable activities. The purpose of this article is to review lectins with anti-HIV activity.

## 2. Lectins

Lectins are carbohydrate-binding proteins of non-immunoglobulin nature. They are capable of recognition of and reversible binding to complicated glycoconjugates moieties without altering the covalent structure of any of the recognized glycosyl ligands [6]. In Nature, lectins are distributed across a wide variety of organisms like algae, fungi, sea corals, higher plants, prokaryotes, invertebrates and vertebrates. They are involved in many biological processes like recognition and binding of carbohydrates, host-pathogen interactions, cell targeting, cell-cell communication and induction of apoptosis, cancer metastasis and differentiation [6]. The antiviral, antifungal and antibacterial activities of lectins have been reported [7,8]. The intent of this article is to review lectins with anti-HIV activity.

## 3. HIV

The human immunodeficiency virus (HIV) type 1 is an enveloped virus that causes the acquired immunodeficiency syndrome (AIDS). In humans the condition is manifested by progressive failure of the immune system [5,9]. It infects viral cells such as T-helper cells, macrophages and dendritic cells [10]. This infection leads to low levels of CD4+ through a number of mechanisms. Also, apoptosis of uninfected cells, direct viral killing of infected cells and killing of infected CD4+ cells by cytotoxic lymphocytes occur. The envelope protein complex determines viral tropism and facilitates the membrane fusion process that allows invasion of the viral genome [11].

## 4. Glycoproteins GP160, Gp120 and Gp41

GP160 is a precursor *env* gene code that is later processed by a host cell protease to form the cleavage products gp120 and gp41. The envelope protein complex and the gp160 are extensively glycosylated and proteolytically cleaved. When new virus particles come to the host cell, gp120 and gp41 will lie on opposite sides of the virus membrane. Gp120 will sit on the outside of the virus particle, forming the virus’s spikes and gp41 will sit on the inside of the membrane. Each gp41 will be anchored to a gp120 through the membrane [12,13].

Gp120 binds to CD4 receptor on any target cell that has such a receptor and will present to T-lymphocytes and macrophages. This CD4 is associated with spontaneous mutation in the *env* gene. The presence of CXCR_4_ is enough for this mutant strain to infect human cells. These strains have seven mutations in the sequence and coding for gp120. It is also proposed that the mutations induce conformational changes in gp120 that allow the virus to directly interact with CXCR_4_ [14]. Prior to binding the host cell, gp120 remains effectively hidden from antibodies. Gp120 is buried in the protein and shielded by sugars, which makes neutralization by antibodies very difficult. It is only exposed when in close proximity to a host cell and the space between the viral and host cell membranes is small enough to hinder the binding of antibodies. This is one of the reasons why there is not a vaccine available to HIV [15]. Gp41 is non-covalently bound to gp120 because it is buried within the viral envelope. When gp120 binds to CD4, it will change its conformation and gp41 will become exposed, where it can assist in fusion with the host cell [12,13].

## 5. Different Lectins

Some lectins exhibit significant activity against human immunodeficiency virus (HIV) and other viruses with an envelope. This makes them particularly attractive targets for the development as novel antiviral drugs. Cyanovirin-*N* and griffithsin are examples of lectins that can inhibit HIV and other viruses [7,16,17,18]. Virally encoded glycoproteins cover the surfaces of retroviruses such as HIV and many other viruses with an envelope. gp41 and gp120 present on the envelope of HIV, heavily glycosylated with glycans, are estimated to contribute almost 50% of the molecular weight of gp120 [19,20,21]. Agents that strongly and specifically interact with the glycans may disturb the interaction between the cells of the host and the proteins of the viral envelope [22,23].

On the viral surface, sugar-binding proteins can crosslink with glycans and prevent other interactions with the co-receptors. Antiviral lectins can prevent penetration of the host cells by the viruses. They prevent infection by binding to the sugars that adorn the surface of the envelope of HIV [24]. The majority of other current antiviral therapeutics act through inhibition of the viral life cycle. Antiviral lectins are suitable for topical applications and can exhibit lower toxicity than others used for antiviral therapy. The antiviral proteins are often resistant to low pH and high temperatures. Also they are odorless, which are properties favorable for potential drugs [19].

Examples of lectins that exhibit antiviral activity and bind high-mannose carbohydrates are jacalin [25], concanavalin A [26], *Urtica diocia* agglutinin [27], *Myrianthus holstii* lectin, *P. tetragonolobus* lectin [28] and *Narcissus pseudonarcissus* lectin [29]. Lectins are also derived from marine organisms. The natural antiviral products that exhibit the highest activity among the lectins are cyanovirin*-N*, scytovirin, microcystis virdis lectin and griffithsin [7,18,30,31].

## 6. Flowering Plant Lectins

### 6.1. Artocarpus Heterophyllus Lectinjacalin

Jacalin is the major protein from the jackfruit *Artocarpus heterophyllus* seeds. *Artocarpus heterophyllus* in the Moraceae family is an evergreen tree that is grown in various tropical countries in South and Southeast Asia. The seed is large and oblong with a brown tegmen and a slim membranous testa.

Chatterjee *et al*. [32], Roque-Barreira *et al*. [33] and Kondoh *et al.* [34], were among the first who identified jacalin around 1980. Jacalin had the property of binding human IgA with specificity for the IgA1 subclass [32,33,34]. In the recent years various studies have been carried out on jacalin, revealing its importance as a lectin of diverse applications ranging from the isolation of human IgA1 to HIV research.

Jacalin has been reported to have a molecular mass of 65–66 kDa [35]. The seeds of this fruit contain a minor mannose-binding lectin [36]. It is a two-chain lectin consisting of an α-chain of a 133 amino acid residues non-covalently bound to a β-chain of 21 amino acid residues [37]. The glycosylated α-chain has a molecular mass of 15.8 kDa [38]. Sugars which interact with the lectinic-site of jacalin suppress the binding of jacalin to CD4. These findings give some insight into the mechanism by which jacalin inhibits HIV-1 infection of CD4+ cells [39].

### 6.2. Canavalia Ensiformis Lectin (Concanavalin A)

Concanavalin A is a carbohydrate-binding protein isolated from *Canavalia ensiformis* or jack bean. It is a member of the legume lectin family and is used for human nutrition or animal fodder [40]. The beans of this plant are mildly toxic and copious consumption should be avoided.

Concanavalin A was the first lectin that was available on a commercial basis and is widely used in biology and biochemistry to characterize glycoproteins and sugar-containing entities on the surface of various cells [41]. The plant contains large quantities of the enzyme urease. This is a reason that the plant cannot be used in fodder mixtures that contains urea. It will liberate harmful ammonia from urea. Because of this, the plant has been investigated as a potential source of the urease enzyme [42].

Concanavalin A is a homotetramer with a molecular mass of 26.5 kDa [43]. It has 235 amino acid residues and binds specifically to certain structures that are found in several glycoproteins, sugars and glycolipids, especially internal and non-reducing terminal α-d-mannosyl and α-d-glucosyl groups [44].

Con A binds to envelope glycoprotein gp120 of HIV-1 and HIV-2 [45]. However, HIV-1 resistant strains have been reported [46]. The detailed mechanism of lectin-mediated neutralization has not been completely elucidated, but there is a minimum of two mechanisms, a direct one and an indirect mechanism to account the efficacy of removal of only a few glycans to render HIV resistant. The direct mechanism is that the mutated *N*-glycosylation sites harbor the “key target glycans” for lectin-mediated neutralization and that these are removed by the mutations. Binding of lectins to these glycans either prevent HIV binding to receptors on the cells or affect alterations of gp120 following binding. The indirect mechanism suggests that the removal of one or more *N*-glycosylation sites renders *N*-glycans elsewhere more exposed to carbohydrases in the Golgi body and the endoplasmic reticulum. Local changes in utilization of glycosylation site can affect the glycosylation setup and protein folding. Thus, lectin-binding high-mannose glycans on the wild type are probably more highly processed in the lectin-selected strains, leading to a loss of their affinity for the [46].

### 6.3. Galanthus Nivalis (Snowdrop) Agglutinin (GNA)-Related Lectins

GNA-related lectins are nearly related in their carbohydrate-binding activities [47,48,49] and exhibit significant anti-HIV, anti-HSV and antitumor [50] actions. The most GNA-related lectins exhibit mannose-binding activity, while *Polygonatum cyrtonema* lectin [51] and *Ophiopogon japonicas* lectin [52] also display carbohydrate affinity to sialic acid.

Some agents that interact with viral-envelope glycans may compromise the efficient entry of the virus into its susceptible target cells. They do not interfere with the glycosylation enzymes from the cell, but rather act by directly binding to the intact glycans on the envelope. This kind of carbohydrate-binding agents force the virus to delete a part of its glycan shield so that it can escape drug pressure. The result of this can be the initiation of an immune response against uncovered immunogenic envelope epitopes.

The mechanisms of antiviral action of the carbohydrate-binding agents are, firstly, direct antiviral activity by binding to the glycans of the viral envelope and blocking entry of the virus and secondly, an indirect antiviral action. This will result from the progressive creation of deletions in the glycan shield of the envelope. The immune system will be triggered to act against previously hidden immunogenic epitopes of the viral envelope [53]. Agglutinins from *Hippeastrum hybrid* and *Galanthus nivalis* inhibited cell-cell fusion between Clone69TRevEnv cells induced to express the viral envelope proteins, gp120/gp41 (Env), and highly CD4-positive SupT1 cells [54].

The mannose-specific plant lectins *Hippeastrum hybrid* agglutinin, *Galanthus nivalis* agglutinin, *Narcissus pseudonarcissus* agglutinin; *Cymbidium* agglutinin; cyanovirin*-N*; and *N*-acetylglucosamine specific *Urtica dioica* agglutinin efficiently abrogate dendritic cell-specific intercellular adhesion molecule-3-grabbing nonintegrin (DC-SIGN)-directed HIV-1 capture and subsequent transmission to T lymphocytes [55]. The mannose-specific plant lectins from *Galanthus*, *Hippeastrum*, *Narcissus*, *Epipactis helleborine*, and *Listera ovata*, and the *N*-acetylglucosamine-specific *Urtica dioica* lectin would primarily be targeted at the virus-cell fusion process [56].

### 6.4. Musa Acuminata (Banana) Lectin

Banana lectin binds to the glycosylated viral envelope and inhibits cellular entry and thus suppresses HIV-1 infection. Its anti-HIV activity is comparable to snowdrop lectin and Griffithsin, and to the anti-HIV drugs T-20 and maraviroc [57].

### 6.5. Myrianthus Holstii Lectin

*Myrianthus holstii* is a tree with a large of branches then can grow from 1 to 20 meters in height. It is of the Urticaceae family and often has stilt roots up to 60 cm high and a short bole. The plant is harvested from the wild as local source of food and wood. This tree is grown in middle Africa, in rainforest, montane forests, at edges or in regrowth and along rivers [58]. *Myrianthus holstii* lectin obtained from the roots by LH-20 chromatography has a molecular mass of 9 kDa. This lectin consists of two major constituents with a molecular mass of 9284 and 9300 Da, respectively. *Myrianthus holstii* lectin protected CEM-SS cells from the cytopathic effects of HIV with an EC_50_ value of 1.4 μg/mL (150 nM). This one is similar to the *Urtica diocia* agglutinin (EC_50_ = 105 nM). It inhibited syncytium formation with an EC_50_ value of 9.8 μg/mL indicating that this lectin reversibly inhibits HIV infection [28].

### 6.6. Narcissus Pseudonarcissus Lectin

*Narcissus pseudonarcissus* is also known as wild daffodil or Lent lily and belongs to the Amaryllidaceae family. This plant produces seeds and is native to Western Europe. It grows in woods, grassland and rocky ground. It contains a dimeric protein with a molecular mass of 26 kDa. It is composed of two 13-kDa subunits. It has specificity toward α-linked mannose and is used in characterization of early stages of apoptosis and has mitogenic activity toward human lymphocytes [59]. This lectin agglutinates rabbit erythrocytes but is non-reactive with human erythrocytes [60].

Mannose-binding agglutinins from other *Narcissus* species also manifest HIV-1 infection inhibitory activity like that of *Narcissus pseudonarcissus* agglutinin. Their HIV-1 infection inhibitory activity does not correlate with their hemagglutinating activity [61].

### 6.7. Polygonatum Cyrtonema Lectin

Comparative analyses revealed that the dimer-based super-structure may be important to the anti-HIV property of *Polygonatum cyrtonema* lectin, a novel anti-HIV mannose-binding lectin from *Galanthus nivalis* agglutinin-related lectin family [62].

### 6.8. Urtica Diocia Agglutinin

*Urtica diocia* is a herbaceous perennial flowering plant and is often called stinging nettle or common nettle. It grows in Europe, North America, Africa and Asia and will not grow more than 2 meters in height. It has a long history of use as a source of fiber, food and medicine. *Urtica diocia* agglutinin is a special plant lectin that differs from other plant lectins. The cytopathic effect of HIV has a rate of EC_50_ = 105 nM [28] and has a small molecular mass of 8.5 kDa. This is a T cell mitogen distinguishable from classical T cell lectin mitogens. It is able to discriminate a particular population of CD4+ and CD8+ cells, and can induce an original pattern of T cell cytokine production as well as activation. Plant lectin mitogens have been successfully and extensively used in the analysis of events that occur during lymphocyte activation and proliferation [63,64].

## 7. Algal Lectins

### 7.1. Boodlea Coacta Lectin

The lectin suppressed entry of HIV-1 into host cells with a half-maximal effective concentration at 8.2 nM. Surface plasmon resonance analysis disclosed a high association constant of the lectin with gp120. The lectin binds to viral envelope hemagglutinin against various strains, including a clinical isolate of pandemic H1N1-2009 virus, indicating that it is potentially useful for antiviral therapy [65].

### 7.2. Griffithsin

Griffithsin is a 13-kDa protein with 121 amino acids isolated from the red alga *Griffithsia*. This protein is a highly potent HIV entry inhibitor [18]. It blocks CD4-dependent GP120 binding to the receptor expressing cells. There it will bind to other gp’s like gp120, 160 and 41. This will happen in a glycosylation-dependent manner. It inhibits gp120 binding of monoclonal antibody (mAb) 2G12, which recognizes a carbohydrate-dependent motif, and (mAb) 48d, which binds to CD4-induced epitope. Griffithsin also moderately interfered with the binding of gp120 to sCD4.

The binding of griffithsin to soluble gp120 was not inhibited by fucose, xylose, galactose, *N*-acetylgalactosamine or sialic acid containing glycoproteins but it was inhibited by mannose, glucose and *N*-acetylglucosamine. Griffithsin binds the terminal mannose residues of surface *N*-linked glycans on HIV-1, HIV-2, Ebola virus, hepatitis C virus, and severe acute respiratory syndrome coronavirus. It means that this is a type of lectin that binds to several viral glycoproteins in a monosaccharide-dependent manner. Griffithsin interacts with gp120 leading to exposure of the CD4-binding site CD4bs through binding of the glycan at position 386 [66]. Unlike cyanovirin and *Galanthus nivalis* agglutinin, the interaction between griffithsin and gp120 relies on the specific trimeric “sugar tower” including N295 and N448. This was supported by findings from Griffithsin Env binding experiments [67].

Griffithsin does not have mitogenic activity toward human T-cells and does not elicit secretion of proinflammatory cytokines in treated human cell lines. Subutaneous injections of griffithsin induce no toxicity other than some splenomegaly and hepatomegaly. Serum samples of griffithsin-treated animals display activity against HIV-1-enveloped pseudoviruses in a cell-based neutralization assay. Griffithsin suppresses HIV gp120 binding to DC-SIGN and DC-SIGN-mediated transfer of HIV-1 to CD4+ T-lymphocytes. It drives gp120 away from the gp120-DC-SIGN complex. Functionally intact carbohydrate binding sites are essential to its optimal action. Its dimeric form is paramount to its HIV inhibitory action. Griffithsin synergizes with the antiviral drugs enfuvirtide, maraviroc and tenofovir against HIV-1 clade B and clade C isolates in primary peripheral blood mononuclear cells and in CD4+ MT-4 cells. Dissimilarity in glycosylation patterns on the viral envelope of clade B and clade C gp120 does not affect the synergism [68].

However, a discrepancy exists between HIV-1 gp120 binding activity and HIV inhibitory activity of the griffithsin variants D30A, D70A, and D112A, suggesting the presence of an anti-HIV mechanism unrelated to simple gp120 binding [69].

Griffithsin has three nearly-equivalent mannose binding sites all essential for anti-HIV activity. Griffithsin mutants D30A, D112A, and D70A, each with mutation of one of the three sites, demonstrated only a 2–3 fold decrement in binding to immobilized gp120 but 26- or more fold decline in HIV inhibiting ability in single-round HIV infection assays, HIV cytopathicity assays and co-cultivation assays. D70A exhibited superior HIV inhibitory activity despite reduced gp120 binding activity compared with D30A and D112A in some strains, furnishing evidence for absence of a direct correlation between binding to HIV gp120 and HIV inhibition. Hence gp120 binding does not necessarily ensue in an antiviral effect; The anti-HIV mechanism of griffithsin action involves not only binding and sterically hindering the activity of gp120, but more importantly to alter the gp120 structure or its oligomeric state as a consequence of the binding of g120 mannose residues, and cross-link gp120 glycans, to render HIV uninfective [69].

Griffithsin interacts with gp120 leading to exposure of the CD4-binding site CD4bs through binding of the glycan at position 386 [66]. Unlike cyanovirin and *Galanthus nivalis* agglutinin, the interaction between griffithsin and gp120 relies on the specific trimeric “sugar tower” including N295 and N448. This was supported by findings from Griffithsin Env binding experiments [67]. However, a discrepancy exists between HIV-1 gp120 binding activity and HIV inhibitory activity of the griffithsin variants D30A, D70A, and D112A, suggesting the presence of an anti-HIV mechanism unrelated to simple gp120 binding [69].

Griffithsin synergizes with other carbohydrate-binding agents in inhibitory action against HIV-1, HIV-2, and even against HIV-1 strains demonstrating resistance to some carbohydrate-binding agents. The carbohydrate-binding agents tested have different binding patterns on the gp120 envelope and hence no steric hindrance. The observation opens up the possibility of employing combinations of carbohydrate binding agents in topical microbicidal agents to counter HIV transmission [70].

### 7.3. Oscillatoria Agardhii Agglutinin (OAA)

A mannose-binding lectin from the green alga *Oscillatoria agardhii* (OAA) with selectivity for the cluster of á1-2-mannose, its homologue *Oscillatoria agardhii* agglutinin homologue (OAAH), and a designed hybrid OAAH (OPA), inhibit HIV replication, syncytium formation between HIV-1-infected and uninfected T cells, DC-SIGN-mediated HIV-1 capture and transmission to CD4+ target T cells, infectivity of a variety of HIV-1 and HIV-2 clinical isolates. Surface plasmon resonance analysis and flow cytometry revealed that both are competitive inhibitors of the binding of the Manα(1–2)Man-specific 2G12 monoclonal antibody. The HIV-1 NL4.3 (2G12res), NL4.3 (MVNres) and IIIB (GRFTres) strains were inhibited as well as the wild-type HIV-1 strains. OAA and OPA synergize with *Hippeastrum hybrid* agglutinin, and griffithsin [71]. combinations of GRFT and other CBAs showed synergistic activity against HIV-1, HIV-2, and even against certain CBA-resistant HIV-1 strains. The CBAs tested appear to have distinct binding patterns on the gp120 envelope and therefore do not necessarily compete with each other’s glycan binding sites on gp120. As a result, there might be no steric hindrance between two different CBAs in their competition for glycan binding (except for the HHA/GNA combination). These data are encouraging for the use of paired CBA combinations in topical microbicide applications (e.g., creams, gels, or intravaginal rings) to prevent HIV transmission [72]. Combined NMR and crystallographic results provide structural insights into the mechanism by which OAA specifically recognizes the branched Man-9 core, distinctly different from the recognition of the D1 and D3 arms at the non-reducing end of high-mannose carbohydrates by other antiviral lectins [73].

## 8. Cyanobacterial Lectins

### 8.1. Cyanovirin-N (CVN)

Cyanovirin*-N* is a bacterial protein that is produced by the cyanobacterium *Nostoc ellipsosporum* that shows virucidal activity against several viruses [7,74]. It is an entry inhibitor of HIV [75]. It was discovered during a screening program in the search for naturally occurring virucidal agents that may be developed into anti-HIV microbicides. This protein consists of a single 101 amino acid chain, which exhibits significant internal sequence duplication. It reveals 13 conservative amino acid changes as well as direct homology between 16 amino acid residues. The molecular mass is approximately 10 kDa [7]. It also consists of four cysteines, which can form two intrachain disulfide bonds.

The intact disulfide bonds appear to be required for anti-HIV activity. When cleavage of these bonds happens due to β-mercaptoethanol treatment its HIV inhibitory effects were abolished. When stored in solution phase, reduced cyanovirin-*N* could reform the required disulfide bonds, accompanied by recovery of anti-HIV activity [75].

Cyanovirin*-N* inhibited the *in vitro* cytopathicity of laboratory strains and several clinical isolates of HIV type 1, 2 and simian immunodeficiency virus. It also effectively prevented cell-to-cell fusion and transmission of HIV from infected cells to uninfected cells. HIV virions that have undergone pretreatment with cyanovirin*-N* neutralized virus infectivity, but are nontoxic to host cells. The unique virucidal effects arise from its association with the viral envelope glycoprotein 120. Cyanovirin*-N* interferes with critical interactions between viral gp120 and cell surface receptors, which are required for successful virus fusion and entry into cells [7].

Linker-cyanovirin*-N* (a cyanovirin*-N* derivative with a flexible and hydrophilic linker (Gly4Ser)3 at the *N*-terminus), and 10 K PEG-ALD-LCVN (*N*-terminal α-amine of *N*-terminal α-amine of linker- cyanovirin*-N* PEGylated to create 10 K PEG-aldehyde- linker- cyanovirin*-N*), are characterized by the specificity and affinity of cyanovirin*-N* for high mannose *N*-glycans. Linker- cyanovirin*-N* displayed anti-HIV-1 activity with reduscd cytotoxicity on CaT keratinocytes and MT-4 T lymphocytes. 10 K PEG-ALD-LCVN inactivated HIV-1 with markedly decreased cytotoxicity and pronounced cell-to-cell fusion inhibitory activity *in vitro*. The linker-extended cyanovirin*-N* and the mono-PEGylated derivative are thus promising candidates for the development of an anti-HIV-1 agent [76]. Humic acids potentiate the anti-HIV activity of AZT, griffithsin, and cyanovirin [77]. CVN specifically recognizes with nanomolar affinity Man(9)GlcNAc(2) and the D1D3 isomer of Man(8)GlcNAc(2) [78].

### 8.2. Scytovirin (SVN)

Scytovirin is an antiviral protein isolated from the cyanobacterium *Scytonema varium.* This protein consists of a single 95-amino acid chain with a molecular mass of 9.7 kDa and significant internal sequence duplication. It has 10 cysteines forming five intrachain disulfide bonds [30]. SVN contains two homologous domains, one comprising amino acids 1–48 and the second amino acids 49–95. SD1 displayed anti-HIV activity analogous to while SD2 demonstrated weaker activity than full-length SVN. Further deletion mutants of the SD1 domain (SVN(3–45)Cys7Ser, SVN(6–45)Cys7Ser, SVN(11–45)Cys7Ser) revealed the importance of *N*-terminal residues for anti-HIV activity of SD1. Deletion of the eight *C*-terminal (SVN(1–40)Cys7Ser) amino acids markedly reduced the anti-HIV activity of SD1. There are two carbohydrate-binding sites with different affinities [30,79]. CVN specifically recognizes with nanomolar affinity Man(9)GlcNAc(2) and the D1D3 isomer of Man(8)GlcNAc(2) [80,81,82].

### 8.3. Microcystis Viridis Lectin

*Microcystis viridis* is a unicellular freshwater bloom-forming cyanobacterium. It showed transient hemagglutinating activity in laboratory culture during stationary phase under nonaeration conditions. This lectin is composed of a single 13-kDa polypeptide with 113 amino acid residues and two tandemly repeated homologous domains of 54 amino residues.

*Microcystis viridis* lectin binds oligomannosides with sub-micromolar affinities and that two novel carbohydrate recognition domains composed of four non-contiguous regions. The residues make numerous intermolecular contacts with their carbohydrate ligands. This lectin inhibits HIV type 1 envelope-mediated cell fusion with an IC_50_ [83].

### 8.4. Microvirin

The cyanobacterial lectin microvirin is potentially exploitable since it potently inhibits HIV-1 with minimal toxicity. Synchronized infectivity assays reveal different kinetics of HIV-1 entry inhibition by microvirin and cyanovirin. Synergism demonstrated by combinations of the inhibitors in spite of common specificity for Maná(1-2)Man terminals indicate recognition of distinctly different glycans and/or glycan conformations on gp120. Entry assays utilizing amphotropic viruses disclose the inactivity of microvirin, in contrast to the potent inhibitory activity of cyanovirin. In view of the similarity in the carbohydrate-binding site common to microvirin and cyanovirin, it appears that gp120 is a clustered glycan epitope and multivalent-protein interactions applicable to cyanovirin but not to microvirin are needed for inhibition of certain viruses [84].

## 9. Actinomycete Lectin

### Actinohivin

Actinohivin, an actinomycete lectin, is composed of 114 amino acid residues and contains three segments, all of which display high specific affinity to gp120 due to multivalent interaction of the three sugar-binding pockets with three high-mannose type glycans HMTGs of gp120 via the “cluster effect” of lectin. It exhibits potent *in vitro* anti-HIV activity [85]. Actinohivin suppresses entry of HIV-1 to susceptible cells. Specific and potent anti-HIV activity is produced by cooperative binding of three segments of actinohivin to three high mannose-type glycans (HMTGs) of HIV-1 gp120. The anti-HIV activity of actinohivin is enhanced after dimerization due to a rise in the number of HMTG-binding pockets [86].

## 10. Worm Lectins

### 10.1. Chaetopterus Variopedatus Polychaete Marine Worm Lectin

The 30-kDa â-galactose-specific lectin inhibited the cytopathic effects brought about by HIV-1 and the production of viral p24 antigen. Its effect at the early stage of viral replication was disclosed by time-of-addition analysis of its anti-HIV-1 activity indicated. The lectin inhibited cell-to-cell fusion of HIV infected and uninfected cells. Its suppression of HIV-1 entry into host cells was shown by employing fluorescence-based real-time quantify PCR [87].

### 10.2. Serpula Vermicularis Sea Worm Lectin

The lectin inhibited the production of viral p24 antigen and cytopathic effect induced by HIV with EC_50_ values of 0.23 and 0.15 μg/mL, respectively [88].

### 10.3. C-Type Lectin Mermaid from Nematode (Laxus Oneistus)

Dendritic cells (DCs) play a vital role in HIV-1 transmission; DCs capture assaulting HIV-1 via interaction of the C-type lectin DC-SIGN with gp120 oligosaccharides and translocate to lymphoid tissues in which transmission of HIV-1 to T cells occurs. It is reasonable to target gp120 for inhibiting interactions with DCs and transmission of HIV-1. The nematode (*Laxus oneistus*) Ca^2+^-dependent C-type lectin Mermaid, a structural homologue of DC-SIGN devoid of cytotoxicity, shares the glycan specificity with DC-SIGN, interacts with high mannose structures on gp120 and prevents HIV-1 binding to DC-SIGN on DCs. Mermaid inhibits DC-SIGN-mediated HIV-1 transmission from DC to T cells. Thus the lectin has potential for development into an anti-HIV-1 agent [89].

## 11. Pradimicins and Benanomicins

Pradimicins and benanomicins are non-peptidic lectin mimics which recognize d-mannopyranoside in the presence of calcium ions. They exert antifungal and anti-HIV activities through binding to mannopyranoside-containing glycans of pathogens. Pradimicin A, an original member of pradimicins, binds to terminal d-mannopyranoside residues at the non-reducing end of glycans, as well as to internal 6-substituted mannopyranoside residues [90].

## 12. Human Mannose Binding Lectin

Mannose binding lectin triggers the complement pathway culminating in pathogen opsonization and phagocytosis. Mannose binding lectin deficiency is associated with to HIV transmission and progression of AIDS. Expression of the lectin has been detected in astrocytes, microglia, neurons, and oligodendrocytes of the HIV-1-infected frontal cortex. In HIV encephalitis, monomeric and trimeric mannose binding lectin expression and MCP-1 expression are elevated in the neuronal axons. Differential MBL expression was not observed in healthy HIV-negative subjects and normal or gp120 transgenic mice. The findings indicate that mannose binding lectin could cause neuroinflammation and neuronal injury through activating the complement pathway [91].

HIV-1goes into the brain at the early stages of infection and leads to damage and impairment of the central nervous system Mannose binding lectin binds to the *N*-linked mannose residues on gp120, a neurotoxin, and is instrumental in intravesicular packaging of gp120 in neuronal subcellular organelles and subcellular trafficking of gp120 through the endoplasmic reticulum and Golgi vesicles.The vesicular complexes were translocated along the microtubule network A functional carbohydrate recognition domain necessary for the actions of mannose binding lectin [92].

## 13. Further Perspectives

In summary, the use of lectins extracted from various sources is a great promise and potential for use in future HIV therapy. Latest research has found potent results towards the anti-HIV effects of various lectins as shown in this review. There is still no vaccine against HIV. The *N*-linked glycans of gp120 V1 protects the V3 region of oligomeric gp120 from potential neutralizing antibodies [93]. Because of the inventive immunological escape mechanisms of this virus, additional research should be funded in order to understand these mechanisms. Also for the lectins, research should be promoted and funded and aid in the transition to clinical application.

The mannose binding lectins Actinohivin (AH), cyanovirin*-N* (CV*-N*), griffithsin (GRFT), *Microcystis viridis lectin* (MVL), *Oscillatoria agardhii* agglutinin (OAA), scytovirin (SVN), from cyanobacteria and algae display high anti-HIV potencies with nanomolar-picomolar IC_50_ values. They differ in tertiary and quaternary structures, but contain internal repeats within their primary structures. The number of sequence repeats often corresponds to the number of domains and binding sites in each lectin. There is pronounced amino acid sequence homology and also structural resemblance between the domains. Each of the lectins has distinctive characteristics. Some are multimeric and/or manifest domain swapping, whereas others are monomeric. Monomeric CV*-N*, OAA, and SVN have two sugar binding sites; AH and engineered monomeric GRFT possess three sites; domain-swapped CV*-N* and MVL display four sites; and domain-swapped GRFT has six sugar binding sites. The lectins recognize different epitopes on high mannoses. The number of binding sites on the protein and/or the number of epitopes recognized on Man-8/9 correlate with its antiviral activity. They target mannose on gp120 of HIV, inhibiting the conformational change into the active state. The most straightforward delivery mode is introduction into the rectum or vagina, or in multifunctional contraceptive gels or rings, or *in situ* expression by modified commensal bacteria which inhabit the gut or vagina [94]. Engineered vaginal lactobacillus strain has been proposed for mucosal delivery of the human immunodeficiency virus inhibitor cyanovirin*-N* to prevent sexual transmission of the virus [95]. GRFT could be obtained in multigram quantities after extraction from *Nicotiana benthamiana* plants transducted with a tobacco mosaic virus vector expressing the lectin. The employment of *Lactobacillus jensenii* expressing an anti-HIV lectin can curtail the costs of the development of a user-friendly microbicide [96]. The anti-HIV activities of various lectins are summarized in Table 1 and Table 2.

**Table 1:** Anti-HIV activity of algal lectins in different assays.

Algal Lectin	Carbohydrate Specificity	Assay of anti-HIV Activity	EC_50_ or IC_50_	Refs.
Cyanovirin *-N* (CV*-N*)	mannose-binding	HIV-1 X4 laboratory strain in CEM-SS cells	0.1–4.8 nM	[7,96,97]
		HIV-1 X4 and X4/R5 laboratory strain in CEM cells	0.7–5 nM	[96,98]
		HIV-1 X4 laboratory strain in MT-4 cells	4 ng/mL	[46,96]
		HIV-1 X4 laboratory strain in MT-4 cells	16 nM	[96,99]
		HIV-1 X4 laboratory strain in MT-2 cells	0.4–5.8 nM	[7,96,97]
		HIV-2 X4 laboratory strain in CEM-SS cells	2.3–7.6 nM	[7,96,97]
		HIV-2 X4 laboratory strain in CEM cells	2 nM	[96,98]
		HIV-1 X4 and R5 laboratory strains in PBMC and macrophages	14–160 nM	[96,98]
		HIV-1 X4 and R5 primary isolate in PBMC and macrophages	0.3–160 nM	[7,96,97,98,100]
		HIV-2 X4 laboratory strain in PBMC	33 nM	[96,98]
		Env-pseudotyped X4, R5 and X4/R5 HIV1 strains in TZM-bl cells	0.1–2 nM	[84,96]
		Env-pseudotyped HIV-1 isolates of clades A/B/C in TZM-bl cells	0.4–18 nM	[96,100]
		SIV in CEM × 174 cells, MT-4 cells or PBMC	11–160 nM	[7,96,98]
microvirin (MVN)	mannose-binding	HIV-1 X4 laboratory strain in MT-4 cells	6 nM	[96,99]
		HIV-2 laboratory strain in MT-4 cells	>262 nM	[72,96]
		HIV-1 X4 and R5 laboratory strains in PBMC	8–22 nM	[96,99]
		HIV-1 clinical isolates (group M) in PBMC	2–167 nM	[96,99]
		HIV-1 clinical isolates (group O) in PBMC	>350 nM	[96,99]
		HIV-2 clinical isolate in PBMC	>350 nM	[96,99]
		Env-pseudotyped X4, R5 and X4/R5 HIV-1 strains in TZM-bl cells	2–12 nM	[96,99]
*Microcystis viridis* lectin (MVL)	mannose-binding	HIV-1 X4 and R5 Env-mediated fusion in a quantitative vaccinia virus reporter gene assay	30–37 nM	[96,101]
Scytovirin (SVN)	mannose-binding	HIV-1 X4 laboratory strain in CEM-SS cells	0.3–7 nM	[30,96,102]	
		HIV-1 X4 and R5 primary isolate in PBMC or macrophages	0.4–393.5 nM	[30,96,100]	
		Env-pseudotyped HIV-1 isolates of clades A/B/C in TZM-bl cells	6.2–187 nM	[96,100]	
*Oscillatoria agardhii* agglutinin (OAA)	Mannose binding	HIV-1 X4 laboratory strain in MT-4 cells	44.5 nM	[96,103]	
Griffithsin (GRFT)	Man/Glc-specific	HIV-1 X4 laboratory strain in CEM-SS cells	0.04 nM	[18,96]	
		HIV-1 X4 laboratory strain in MT-4 cells	0.1–0.21 nM	[68,96,104]	
		HIV-1 R5 and X4 strains in MAGI cells	0.03–0.15 nM	[96,105]	
		HIV-2 laboratory strain in MT-4 cells	0.11–0.24 nM	[72,96]	
		HIV-1 X4 and R5 laboratory strains in PBMC	0.16–0.28 nM	[68,96,105]	
		HIV-1 X4 and R5 primary isolate in PBMC or macrophages	0.05–47.6 nM	[18,68,96,100,105]	
		Env-pseudotyped HIV-1 R5 strains in TZM-bl cells	0.02–0.04 nM	[96,105]	
		Env-pseudotyped HIV-1 isolates of clades A/B/C in TZM-bl cells	<3–150 ng/mL	[66,96]	
		Env-pseudotyped HIV-1 isolates of clades A/B/C in TZM-bl cells	0.1–56 nM	[96,100]	
		SIV and SHIV in CEM × 174 cells	0.95–1.24 nM	[96,104]	
		SHIV and R5 HIV-1 in PBMC	0.02–0.04 nM	[96,104]	
		SHIV in MOLTCCR5 cells	0.83 nM	[96,104]	

EC_50_ or IC_50_: concentration required to inhibit virus replication by 50%.

**Table 2:** Anti-HIV activities of lectins from marine invertebrate animals and a marine alga.

Lectin	Carbohydrate Specificity	Anti-HIV Activity	EC_50_	Ref.
Griffithsin from Red algae *Griffithsia* sp	Man/Glc-specific lectin	Against T cell tropic and macrophage tropic strains of HIV-1	Ranging from 0.043–0.63 μM	[106,107]
		Abort cell-to-cell fusion and transmission of HIV-1 infection	Ranging from 0.043–0.63 μM	
Lectin from marine worm *Chaetopterus variopedatus* (CVL)	β-galactose-specific lectin	Inhibit HIV-induced syncytium formation	0.0043 μM	[87,107]
		Inhibit HIV-1 p24 production	0.057 μM	
Lectin from marine worm *Serpula vermicularis* (SVL)	GlcNAc-specific lectin	Inhibit HIV-induced syncytium formation	0.15 μg/mL	[88,107]
		Inhibit HIV-1 p24 production	0.23 μg/mL	
Lectin from marine mussel *Crenomytilus grayanus* (CGL)	High affinity to the glycoproteins of mucin type	Inhibit HIV-replication	45.7 μg/mL	[107,108]
Lectin from Ascidium *Didemnum ternatanum* (DTL)	GlcNAc-specific lectin	Inhibit HIV-replication	0.006 μg/mL	[107,108]
DTL-A from Ascidium *didemnum ternatanum*	GlcNAc/GalNAc and heparin-binding lectin	Inhibit HIV-replication	0.59 μg/mL	[107,108]
Lectin from marine worm *Serpula vermicularis* (SVL-1)	Mannan-binding lectin	Inhibit HIV-replication	89.1 μg/mL	[107,108]
Lectin from marine worm *Serpula vermicularis* (SVL-2)	GlcNAc-specific lectin	Inhibit HIV-replication	0.23 μg/mL	[107,108]

EC_50_ = 50% effective concentration.
